# A case of repeated stent fracture

**DOI:** 10.1093/omcr/omae075

**Published:** 2024-07-21

**Authors:** Shohei Migita, Nobuhiro Murata, Kurara Takahashi, Yuki Nakajima, Saki Mizobuchi, Masatsugu Miyagawa, Yudai Tanaka, Katsunori Fukumoto, Riku Arai, Tomoyuki Morikawa, Takashi Mineki, Keisuke Kojima, Mitsumasa Sudo, Daisuke Fukamachi, Yasuo Okumura

**Affiliations:** Division of Cardiology, Department of Internal Medicine, Nihon University School of Medicine, Ohyaguchi-kamicho, Itabashi-ku, Tokyo 173-8610, Japan; Division of Cardiology, Department of Internal Medicine, Nihon University School of Medicine, Ohyaguchi-kamicho, Itabashi-ku, Tokyo 173-8610, Japan; Division of Cardiology, Department of Internal Medicine, Nihon University School of Medicine, Ohyaguchi-kamicho, Itabashi-ku, Tokyo 173-8610, Japan; Division of Cardiology, Department of Internal Medicine, Nihon University School of Medicine, Ohyaguchi-kamicho, Itabashi-ku, Tokyo 173-8610, Japan; Division of Cardiology, Department of Internal Medicine, Nihon University School of Medicine, Ohyaguchi-kamicho, Itabashi-ku, Tokyo 173-8610, Japan; Division of Cardiology, Department of Internal Medicine, Nihon University School of Medicine, Ohyaguchi-kamicho, Itabashi-ku, Tokyo 173-8610, Japan; Division of Cardiology, Department of Internal Medicine, Nihon University School of Medicine, Ohyaguchi-kamicho, Itabashi-ku, Tokyo 173-8610, Japan; Division of Cardiology, Department of Internal Medicine, Nihon University School of Medicine, Ohyaguchi-kamicho, Itabashi-ku, Tokyo 173-8610, Japan; Division of Cardiology, Department of Internal Medicine, Nihon University School of Medicine, Ohyaguchi-kamicho, Itabashi-ku, Tokyo 173-8610, Japan; Division of Cardiology, Department of Internal Medicine, Nihon University School of Medicine, Ohyaguchi-kamicho, Itabashi-ku, Tokyo 173-8610, Japan; Division of Cardiology, Department of Internal Medicine, Nihon University School of Medicine, Ohyaguchi-kamicho, Itabashi-ku, Tokyo 173-8610, Japan; Division of Cardiology, Department of Internal Medicine, Nihon University School of Medicine, Ohyaguchi-kamicho, Itabashi-ku, Tokyo 173-8610, Japan; Division of Cardiology, Department of Internal Medicine, Nihon University School of Medicine, Ohyaguchi-kamicho, Itabashi-ku, Tokyo 173-8610, Japan; Department of Cardiology, Nihon University Hospital, Kanda-surugadai, Chiyoda-ku, Tokyo 101-8309, Japan; Division of Cardiology, Department of Internal Medicine, Nihon University School of Medicine, Ohyaguchi-kamicho, Itabashi-ku, Tokyo 173-8610, Japan

**Keywords:** drug-eluting stent, everolimus-eluting stent, percutaneous coronary intervention, stent fracture

## Abstract

Stent fracture is one of the complications of drug-eluting stent implantation. An 84-year-old man underwent coronary angiography for unstable angina. He had diffuse severe stenosis and calcified plaque in the left anterior descending artery and underwent percutaneous coronary intervention (PCI) in the left anterior descending artery for severe stenosis with chest pain. Thereafter, two subsequent stent fractures occurred, so the patient underwent another PCI to cover them. However, a stent fracture was found again one year later. The patient was asymptomatic and PCI was avoided due to the risk of further stent fracture. When a stent fracture occurs, it is important to provide appropriate treatment according to the anatomical findings of the vessel, symptoms and the presence of ischemia.

## Introduction

The use of drug-eluting stent (DES) is common in percutaneous coronary intervention (PCI) for coronary artery disease. Although DES reduced in-stent restenosis by inhibiting neointimal tissue proliferation, stent fracture, in which the stent tears, has been reported as one of the complications of DES implantation [1]. Stent fracture has become a major concern after DES implantation due to its potential association with in-stent restenosis and stent thrombosis [2]. The present report describes a rare case of repeated stent fracture.

## Case report

**Figure 1:**
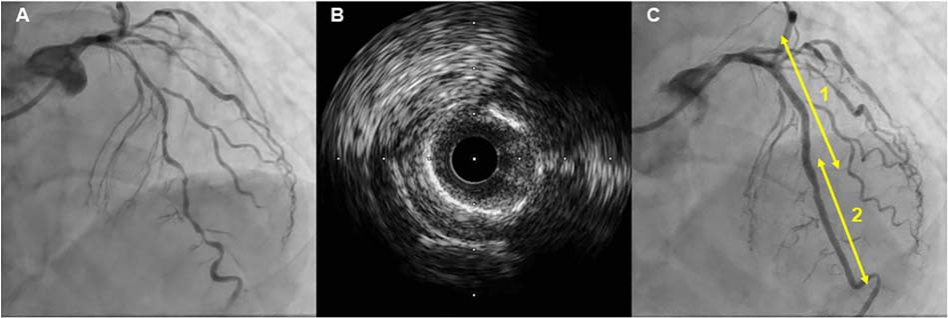
First PCI: Angiogram of LAD showed diffuse severe stenosis (**A**) and calcified plaque on intravascular ultrasound (**B**), and two stents (1; XIENCE Skypoint 3.0 × 48 mm, 2; XIENCE Skypoint 2.25 × 15 mm) were implanted (**C**).

**Figure 2:**
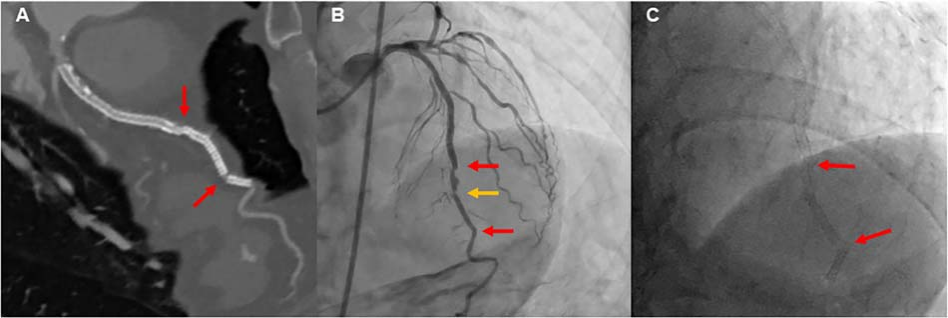
Second PCI: CCTA showed two stent fractures (**A**), and angiogram of LAD also showed two stent fractures and new stenosis (**B** and **C**).

**Figure 3:**
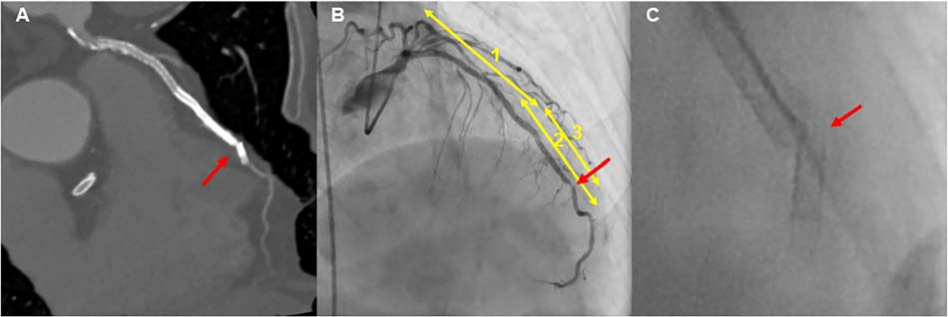
Follow-up CAG: A new stent fracture was confirmed by CCTA (**A**), and angiogram of LAD showed a stent fracture at the stent duplex (1; XIENCE Skypoint 3.0 × 48 mm, 2; XIENCE Skypoint 2.25 × 15 mm, 3; SYNERGY XD 2.25 × 38 mm) (**B** and **C**).

An 84-year-old man underwent coronary angiography (CAG) for unstable angina pectoris. There were diffuse severe stenosis (Fig. 1A) and calcified plaque (Fig. 1B) in the left anterior descending artery (LAD). Due to severe stenosis with chest pain, the patient was deemed eligible for revascularization and underwent PCI. Two everolimus-eluting stents (EES) (XIENCE Skypoint 3.0 × 48 mm, XIENCE Skypoint 2.25 × 15 mm) were implanted after adequate balloon dilatation with scoring balloon (Fig. 1C). Eight months later, the patient had chest pain on exertion and coronary computed tomography angiography (CCTA) revealed two fractures distal to the LAD stent (Fig. 2A). Due to worsening chest pain on exertion, an urgent CAG was performed, which revealed two stent fractures and severe stenosis in multiple locations associated with stent deformity. DES implantation was chosen to cover multiple stenoses, and a new EES (SYNERGY XD 2.25 × 38 mm) was implanted over the two fracture sites (Fig. 2B and C). However, at CCTA one year later, a fracture was found again at the distal portion of the stent over lapping (Fig. 3A). On repeat CAG, the fracture occurred again at the distal site where the stent was doubled due to a previous stent fracture (Fig. 3B and C). As the patient was asymptomatic and the non-hyperemic pressure ratio was negative (diastolic hyperemia-free ratio: 0.95), PCI was avoided due to the risk of further stent fracture.

## Discussion

Stent fracture is one of the complications of DES implantation. Its incidence is estimated to be 1%–30%, and while DES fractures with little or no lumen narrowing can pass asymptomatically, they can cause inadequate drug delivery and in-stent restenosis, in addition to other consequences such as stent thrombosis, acute coronary syndromes including ST-elevation myocardial infarction, and even aneurysm formation [3, 4]. The incidence of stent fracture in clinical practice has increased significantly in recent years due to the development of stents with thinner steels and the widespread use of intraluminal imaging technology [5]. Risk factors for stent fracture include longer stent length, overlapping stent edges, angulated and calcified lesions, sirolimus-eluting stents, right coronary artery lesions, saphenous vein lesions and aggressive dilatation [1, 3, 6]. There is no standardized treatment, and PCI is effective for symptomatic and ischemic lesions, and the use of DES is considered the most reasonable [7, 8]. In the present case, the meandering and calcification of the LAD was relatively severe, and in addition, the length of the lesion was long, which was considered to be a contributing factor to the increased length of the stent. In addition, all three stent fractures in this case were type IV stent fractures in the stent fracture classification proposed by Lee SE et al [2]. This indicates a complete transverse stent fracture with displacement of the fracture fragment by more than 1 mm during the cardiac cycle. Type IV stent rupture is easily detected but does not necessarily cause significant restenosis [2]. Therefore, the response to calcified lesions and the assessment of ischemia in this case should also be reviewed to avoid repeat stent fractures. Specifically, in this case, PCI was chosen for severe stenosis because the patient was symptomatic, but detailed ischemic assessment was necessary to determine stent location and indication for a second PCI. Additionally, the use of debulking devices such as rotational atherectomy should be considered for severely calcified lesions during the first PCI. Furthermore, for the second PCI, patients had to consider the option of balloon dilatation alone or drug-coated balloon for the risk of further stent fracture. After the second stent fracture, the patient is asymptomatic. We explained to the patient that when chest pain appears and ischemia is detected, PCI with DCB or revascularization with CABG should be considered.

## Conclusion

Cases of not only repeated stent fracture but also fracture of the overlapping portion of the stent are extremely rare. When a stent fracture occurs, it is important to provide appropriate treatment based on the anatomical findings of the vessel, the symptoms and the presence of ischemia.

## Ethical approval

This case report did not require review by the relevant ethics committees.

## Consent

Written informed consent was obtained from the patient for publication of this case report.

## Gurantor

All the authors are nominated guarantors of the manuscript.
